# Does exposure to malaria prevention messages influence insecticide-treated net use through knowledge of malaria? Evidence from a causal mediation analysis among women in Uganda

**DOI:** 10.3389/fpubh.2026.1948476

**Published:** 2026-10-21

**Authors:** Edson Mwebesa, Delight Mawufemor Agbi, Charles Natuhamya, Benson Musinguzi, Jonathan Izudi, Gregory Kerich, Ann Mwangi

**Affiliations:** 1 Department of Mathematics, Physics and Computing, School of Science and Aerospace Studies, Moi University, Eldoret, Kenya; 2 Department of Mathematics, Muni University, Arua, Uganda; 3 Uganda Youth Development Link, Kampala, Uganda; 4 Department of Medical Laboratory Sciences, Muni University, Arua, Uganda; 5 Department of Immunology and Molecular Biology, School of Biomedical Sciences, College of Health Sciences, Makerere University, Kampala, Uganda; 6 Directorate of Graduate Training, Research and Innovation, Muni University, Arua, Uganda; 7 Department of Community Health, Mbarara University of Science and Technology, Mbarara, Uganda

**Keywords:** causal mediation analysis, health communication, insecticide-treated nets, malaria, Uganda

## Abstract

Exposure to malaria prevention messages is associated with increased use of insecticide-treated nets, but the mechanisms remain unclear. This study assessed whether comprehensive malaria knowledge mediates the relationship between exposure to malaria prevention messages and insecticide-treated net (ITN) use among women of reproductive age in Uganda. We analyzed data from women aged 15–49 years from the 2018/2019 Uganda Malaria Indicator Survey (UMIS). Using a counterfactual causal mediation framework, the study decomposed the total effect (TE) of exposure to malaria prevention messages into the natural direct effect (NDE) and the natural indirect effect (NIE), adjusting for socio-demographic confounders to identify whether malaria knowledge mediated the effect of exposure to malaria prevention messages on ITN use. Among 8,868 participants (8,231 non-refugee women, 637 refugee women), 6,143 (69.3%) reported using an ITN on the night before the survey, 3,334 (37.6%) had been exposed to malaria prevention messages, and 5,427 (61.2%) had comprehensive knowledge of malaria. Exposure to malaria prevention messages significantly increased ITN use by 6.5 percentage points TE = 0.065, 95% confidence interval [CI]: 0.034, 0.094, *p* < 0.001. The NDE of malaria prevention message exposure on ITN use remained significant [0.064, 95% CI: 0.034, 0.092, *p* < 0.001], while the NIE of malaria prevention messages through comprehensive malaria knowledge was not statistically significant [0.001, 95% CI: −0.002, 0.005, *p* = 0.462], accounting for approximately 1.5% of the total effect. Exposure to malaria prevention messages is associated with increased ITN use primarily through direct behavioral pathways rather than through knowledge acquisition. Communication strategies should therefore integrate motivational and behavioral reinforcement components alongside informational content to maximize public health impact.

## 1 Introduction

Malaria is a leading cause of mortality and morbidity in sub-Saharan Africa and disproportionately affects vulnerable populations, especially women and children under 5 years (1, 2). According to the World Health Organization (WHO) 2025 malaria report, there were 282 million malaria cases globally in 2024, representing a 3% increase compared with 2023 and 22.6% compared with 2015. Of the cases reported in 2024, 88% of the increase occurred in the World Health Organization (WHO) African Region, which accounted for 94% of all global malaria cases (3). In 2023, the global malaria incidence rate was 604 cases per 10,000 people at risk, an increase from 252 cases per 10,000 in 2022 (4). Malaria-related deaths reduced from 627000 deaths in 2020 to 597000 deaths in 2023 (4, 5). Uganda ranked third with the highest malaria burden globally, with nearly 12 million malaria cases and approximately 3000 deaths in 2023 (4) and remained in a similar position in 2024 (3). Uganda remains a malaria-endemic country, with malaria transmission occurring in 96% of the geographical regions (6), and malaria is associated with 30–40% of outpatient visits, 20% of admissions, approximately 10% of inpatient deaths, and a 6% hospitalization rate, four times higher than the global average in 2023 (7, 8). The prevalence of malaria is higher among pregnant women (9) and children under 5 years of age, ranging from 9% to 39% (10, 11). Challenges such as drug resistance, social, behavioral, belief-related, and cultural issues, logistical constraints, delayed diagnosis and treatment, and rapidly evolving mosquito behaviors complicate malaria control efforts (4, 12, 13).

A key intervention in malaria control strategies is the widespread adoption of insecticide-treated nets (ITNs) (12). These nets are one of the most effective and efficient interventions and are easily deployable physical barriers against mosquito bites, helping prevent malaria transmission (14). However, consistent ITN use remains suboptimal (11, 15). Despite global investments targeting malaria control and subsequent efforts, achieving widespread malaria control remains challenging in many high malaria-burden African nations (16, 17). This challenge is worsened by inconsistent utilization of ITNs among vulnerable groups such as women, hindering progress toward global malaria eradication targets (14) to reduce malaria incidence and mortality by 90% by 2030 (5).

Mass media, online platforms, and interpersonal communication campaigns have been widely employed in Uganda to promote ITN ownership and use through social and behavior change communication (SBCC) channels. Evidence from epidemiological studies shows that exposure to malaria prevention messages is positively associated with ITN use (18, 19). Exposure to malaria prevention messages improves knowledge of malaria prevention (20) and ITN use (21) among women. Different sources of malaria prevention messages have also been found to significantly improve ITN use, but effect estimates varied across platforms (22). However, the mechanisms underlying the relationship between exposure to malaria prevention messages (malaria messages) and ITN use through knowledge of malaria are not fully understood. An earlier study (21), using a similar data source and population, estimated the effect of exposure to malaria messages on ITN use using propensity score matching (PSM). This study extends that work by applying causal mediation analysis (CMA) to estimate the direct effect (DE) of message exposure and the indirect effect (IE) through comprehensive malaria knowledge in the full sample. Health behavior theories, such as the Health Belief Model and the Ideation Model of communication and behavior change, posit that knowledge is a critical cognitive mediator that translates messages into health practices (23–25), such as ITN use.

Despite the theoretical importance of malaria knowledge, studies empirically testing its mediating role in the pathway between exposure to malaria messages and ITN use are lacking, particularly those based on nationally representative data and robust causal mediation methods. Prior cross-sectional studies in Uganda and elsewhere have shown an association between malaria knowledge, media exposure, and ITN use (26–28). However, these studies relied on traditional regression adjustment, which precluded assessing the direct and indirect effects of malaria knowledge or addressing potential confounding in mediation pathways. Causal mediation analysis, a causal inference approach, quantifies the extent to which knowledge of malaria mediates the relationship between exposure to malaria messages and ITN use based on a counterfactual framework (29, 30). This study evaluates whether the relationship between exposure to malaria messages and use of ITNs is mediated by knowledge of malaria among women of reproductive age in Uganda. Evidence from this study will inform the design of effective social and behavioral change communication interventions to improve malaria control in high-burden countries in sub-Saharan Africa.

## 2 Methods

### 2.1 Study design and setting

We analyzed the 2018/2019 Uganda Malaria Indicator Survey (UMIS) data, a nationally representative household survey implemented by the National Malaria Control Division (NMCD) and Uganda Bureau of Statistics (UBOS) with technical assistance from the International Classification of Functioning, Disability and Health (ICF) (11). The UMIS data were collected from 15 subregions (320 clusters) and 9 refugee settlements (22 clusters) between December 2018 and January 2019. The 2018/2019 UMIS employed a two-stage stratified cluster sampling design, with enumeration areas as primary sampling units and households selected systematically within each cluster. All eligible women (15–49 years) in selected households were included in the study. This survey collected data on malaria knowledge and opinions, such as causes, treatment, and prevention, as well as exposure to malaria messages and their sources. The survey also collected data on ITN ownership and use, background characteristics such as age, education, marital status, religion, and residence, and other household characteristics from women aged 15–49 years. The sample comprised 8,868 (8231 non-refugee and 637 refugee) women aged 15–49 years from selected households. Detailed information on survey design is published elsewhere (11).

### 2.2 Variables

- **Outcome (Y)**: The outcome of the study is the use of an ITN the night before, coded as 1 if the participant slept under an ITN and 0 otherwise.
- **Exposure (T):** The exposure was whether the individual heard or saw malaria control messages in the 6 months preceding the survey. Exposure was measured on a binary scale, coded as 1 if the individual had the exposure and 0 otherwise.
- **Mediator (M):** The mediator variable was comprehensive knowledge of malaria, measured on a binary scale based on items from the Uganda Malaria Indicator Survey (UMIS) 2020. A value of 1 was assigned to individuals who correctly answered all seven malaria-related questions, and 0 otherwise. The knowledge questions were grouped into three constructs: (1) knowledge of the cause of malaria, measured by (i) correctly identifying mosquito bites (Yes = 1, No = 0) or (ii) mosquitoes as the cause (Yes = 1, No = 0); (2) knowledge of malaria prevention, measured using two items: (iii) whether the respondent was aware that malaria can be avoided (Yes = 1, No = 0) and (iv) whether sleeping under an ITN was the best way to prevent malaria (Agree = 1, Disagree/do not know/uncertain = 0); and (3) knowledge of malaria treatment, measured using three items: (v) whether pregnant women should take antimalarial drugs (Agree = 1, Disagree/don't know (DK)/uncertain = 0), (vi) whether a child with fever should be taken to a health provider the same or next day (Agree = 1, Disagree/do not know/uncertain = 0), and (vii) recommended timing for seeking treatment after a child develops fever (same or next day = 1, otherwise = 0). These items were informed by the 2020 UMIS report (11).
- **Covariates (C)** included age categories (15–19, 20–24, 25–29, 30–34, 35–39, 40–44, 45–49), educational level (none, primary, and secondary/higher), total children ever born (0–1 births, 2–3 births, 4 or more), wealth quintile (poorest, poorer, middle, richer, and richest), sex of household head (male or female), number of household members (1–3, 4–6, 7+), type of residence (urban, rural, and refugees), region (central, eastern, northern, and western). Other covariates included: age of the household head (15–29, 30–39, 40–49, 50–59, 60 or more), number of children under 5 years of age in the household (none, 1–2, 3 or more). We selected these covariates because of their associations with the exposure and outcome, as well as existing literature in sub-Saharan Africa (15, 31–37). ITN access was not included among the covariates because it could be influenced by malaria message exposure and therefore represent a parallel mediating causal pathway to ITN use. Adjusting for ITN access could block part of the total effect (TE) of message exposure and change the estimand of interest.

### 2.3 Causal mediation analysis framework

Traditional regression-based mediation approaches (38–40) have limitations when applied to cross-sectional data because of omitted variable bias and untestable assumptions of no exposure–mediator interaction (30, 41). Therefore, we applied a counterfactual causal mediation framework (29, 42–44). Using this approach, we decomposed the total effect (TE) of exposure to malaria messages (T) on ITN use the night before (Y) into the natural direct effect (NDE) and the natural indirect effect (NIE) through comprehensive malaria knowledge (M). The NDE captures pathways independent of the mediator or not operating through the mediator knowledge, whereas the NIE quantifies the portion of the effect operating through malaria knowledge.

Under the counterfactual framework for causal mediation (30, 42), we used the following notation. Let *T* be the exposure to malaria messages (1 = Yes, 0 = No), *M* be the comprehensive knowledge of malaria (1 = Yes, 0 = No), *Y* be ITN use the night before (1 = Yes, 0 = No), and **C** be the vector of observed confounders. *Y*(*t, m*) is the potential outcome if the exposure were set to *t* and the mediator were set to *m*. *M*(*t*) is the potential mediator value if exposure were set to *t*. The total effect (*TE*) of *T* on *Y* is shown in the equation below.


$$\begin{array}{l} TE=\left[Y\left(1,\;M\left(1\right)\right)-Y\left(0,\;M\left(0\right)\right)\right] \end{array}$$

The average effects were estimated using the equations below.


$$\begin{array}{lll} NDE\; & = & E\left[Y\left(1,\;M\left(0\right)\right)-Y\left(0,\;M\left(0\right)\right)\right]\\ NIE\; & = & E\left[Y\left(1,\;M\left(1\right)\right)-Y\left(1,\;M\left(0\right)\right)\right] \end{array}$$

The total effect was obtained on the odds ratio (OR) scale, as below.


$$\begin{array}{l} OR_{TE}=OR_{NDE}\times OR_{NIE} \end{array}$$

Alternatively, on the log-odds scale, as shown below.


$$\begin{array}{l} \log OR_{TE}=\log OR_{NDE}+\log OR_{NIE} \end{array}$$

Natural direct and indirect effects were estimated using the natural effects model (30, 45). Identification of the natural direct and indirect effects relied on the following no-unmeasured-confounding assumptions (30): (1) no unmeasured confounding of the exposure (T)–outcome (Y) relationship, (2) no unmeasured confounding of the exposure (T)–mediator (M) relationship, (3) no unmeasured confounding of the mediator (M)–outcome (Y) relationship, and (4) no confounder of the mediator (M)–outcome (Y) relationship that is itself affected by the exposure. Assumptions (1) and (2) are plausible because of the covariates included in the UMIS; assumptions (3) and (4) are not directly testable and were assessed through sensitivity analysis.

### 2.4 Statistical analysis

Descriptive statistics including counts and percentages with their 95% confidence intervals (CIs) were used to summarize sample characteristics, exposure status, outcome, and mediator variables. Descriptive analyses accounted for the survey design and sampling weights. This study applied CMA within the counterfactual framework (42) to decompose the total effect (TE) of malaria message exposure on ITN use into the natural direct effect (NDE) and natural indirect effect (NIE) through comprehensive malaria knowledge (the mediator). Two regression models were specified:

1. Mediator Model:
$$\begin{array}{l} logit\left\{P\left(M=1\right)\right\}=f\left(T,\;\text{C}\right) \end{array}$$
2. Outcome Model:
$$\begin{array}{l} logit\left\{P\left(Y=1\right)\right\}=f\left(T,\;M,\;\text{C}\right) \end{array}$$

where *T* is the exposure variable, *M* is the mediator variable, *Y* is the outcome variable (ITN use), and **C** is the set of covariates. Both models were estimated using logistic regression incorporating Malaria Indicator Survey (MIS) sampling weights. The mediator and outcome model results (adjusted odds ratios, 95% confidence intervals, and *p*-values) are presented in the Supplementary Tables S7 and S8, respectively. Cluster-robust inference was implemented using the primary sampling unit (PSU) identifier.

To aid the interpretation of the mediation decomposition, potential outcome means were reported for the four mediation ‘worlds', which are *E*[*Y*(1, *M*(1))], *E*[*Y*(1, *M*(0))], *E*[*Y*(0, *M*(0))] and *E*[*Y*(0, *M*(1))] obtained by Monte Carlo simulation from the fitted mediator and outcome models and averaged using MIS sampling weights. *E*[*Y*(1, *M*(1))] (fully exposed world) is the expected outcome if every woman were exposed and had comprehensive malaria knowledge at the level it would have under exposure). *E*[*Y*(1, *M*(0))] (which isolates the direct effect of exposure) is the expected outcome if every woman is exposed to malaria messages, but the comprehensive malaria knowledge was fixed at the level it would have under no exposure). *E*[*Y*(0, *M*(0))] (fully unexposed world) is the expected outcome if no woman was exposed to malaria messages and the comprehensive knowledge of malaria took its value under no exposure). *E*[*Y*(0, *M*(1))] (which isolates the mediator pathway alone) is the expected outcome if no woman were exposed but the mediator (comprehensive malaria knowledge) was set at a level it would have under no exposure.

The results for NDE and NIE on the odds ratio scale are presented in Supplementary File S1. Sensitivity to unmeasured confounders was assessed using E-values (46, 47), which quantify the minimum strength of association on the risk ratio (RR) scale that an unmeasured confounder would need to have with both exposure and outcome, conditional on observable covariates, to account for the observed exposure–outcome association and move the estimated effect to the null or shift the confidence interval to include the null. Because ITN use was not a rare outcome, odds ratios were converted to approximate RRs before E-value estimation. Analyses were conducted in Stata 19 and RStudio 4.5.2. Sampling weights were applied to ensure the generalizability of the findings to the national population of women. Statistical significance was assessed at the 5% level, and 95% CIs were reported.

Figure 1 shows the DAG representing the assumed causal structure underlying the mediation analysis. Exposure to malaria messages (*T*) may influence ITN use (*Y*) directly or indirectly through malaria knowledge (*M*). Observed covariates (**C**) are assumed to confound the relationships between the outcome, exposure, and mediator and were adjusted for in all models. The analysis assumes no unmeasured confounding of the exposure–outcome, exposure–mediator, or mediator–outcome relationships conditional on **C**. Covariates (**C**) were assumed to affect both *T* and *Y*, creating potential backdoor pathways. These relationships are shown by dotted arrows in the DAG and were controlled for in both outcome and mediator models to satisfy the no-unmeasured-confounding assumption required for identification of NDE and NIE. Path *a* represents the effect of malaria message exposure on comprehensive malaria knowledge, while *b* represents the effect of comprehensive malaria knowledge on ITN use conditional on the exposure. Path *c*′ represents the direct effect of malaria message exposure on ITN use that is not mediated by comprehensive malaria knowledge. The TE of malaria message exposure on ITN use was decomposed as the NDE corresponding to the direct pathway (*T*→*Y*) and the NIE operating through malaria knowledge (*T*→*M*→*Y*). The study hypothesized that the causal pathway was exposure to malaria messages → comprehensive malaria knowledge → ITN use.

**Figure 1:**
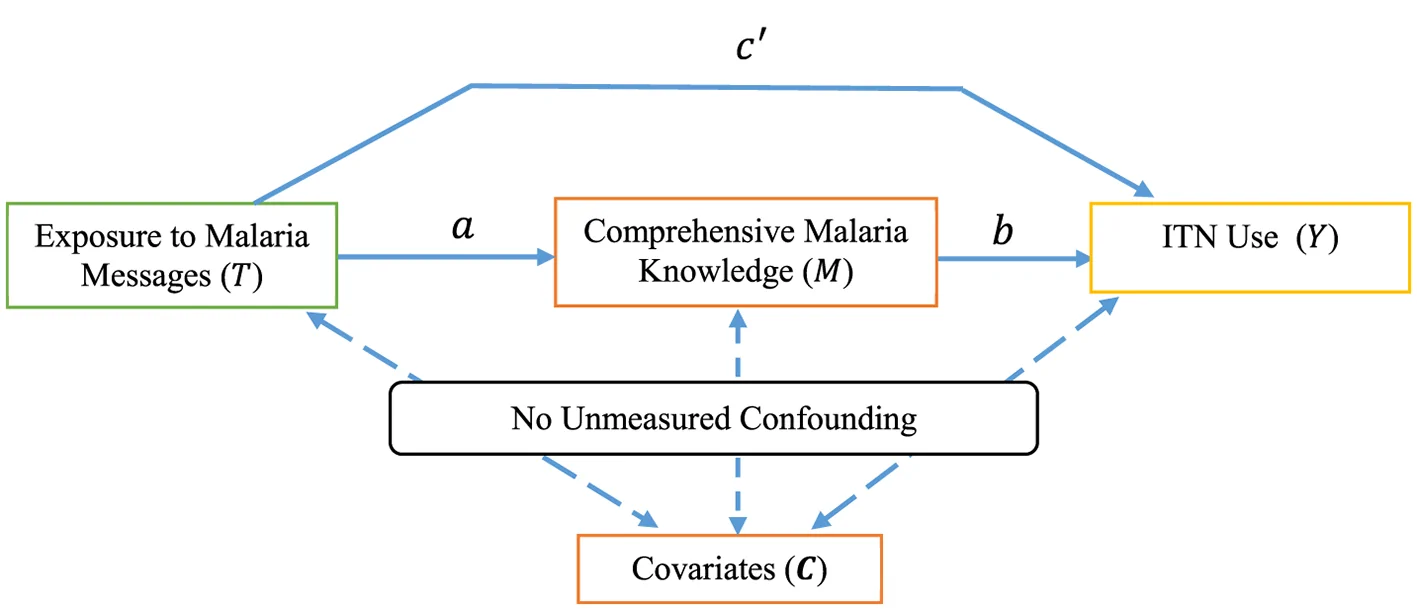
Directed Acyclic Graph (DAG).

## 3 Results

### 3.1 Study participants' characteristics

Of 8,868 participants, 2,037 were aged 15–19 years (23.0%, 95% CI: 21.6–24.4). Women who had primary education were 4584 (51.7%); 3480 (39.2%) had 0–1 births; 2092 (23.6%) lived in the richest wealth quintile; 6129 (69.1%) households were headed by males, of whom 2616 (29.5%) were aged 40–49 years. Households with 1 – 2 children under 5 years of age were 5303 (59.8%), while 3924 (44.2%) of the households had seven or more residents. Women residing in rural areas were 5867 (66.2%), and 2914 (32.9%) were from Western Uganda. Results are shown in Table 1.

**Table 1:** Study sample characteristics (Weighted sample: *n* = 8868).

Covariates	Count	Per cent [95% CI]
Age in 5-year groups		
15–19	2037	23.0 [21.6, 24.4]
20 – 24	1697	19.1 [17.8, 20.5]
25–29	1477	16.7 [15.5, 17.9]
30–34	1281	14.4 [13.4, 15.5]
35–39	1024	11.5 [10.5, 12.6]
40–44	803	9.1 [8.2, 10.0]
45–49	550	6.2 [5.6, 6.9]
Highest education level		
No formal education	1242	14.0 [12.6, 15.5]
Primary	4584	51.7 [48.8, 54.6]
Secondary/higher	3042	34.3 [31.1, 37.8]
Total number of children ever born		
0 - 1 births	3480	39.2 [37.3, 41.2]
2 - 3 births	1950	22.0 [20.8, 23.3]
4 or more births	3438	38.8 [36.3, 41.3]
Wealth Index combined		
Poorest	1824	20.6 [18.4, 22.9]
Poorer	1759	19.8 [17.7, 22.2]
Middle	1527	17.2 [14.9, 19.8]
Richer	1666	18.8 [16.6, 21.2]
Richest	2092	23.6 [18.8, 29.2]
Sex of Household head		
Male	6129	69.1 [66.7, 71.4]
Female	2739	30.9 [28.6, 33.3]
Age of household head		
15–29	1527	17.2 [15.7, 18.8]
30–39	2400	27.1 [25.4, 28.8]
40–49	2616	29.5 [27.5, 31.5]
50–59	1303	14.7 [13.5, 16.0]
60 or more	1023	11.5 [10.2, 13.0]
Number of children 5 and under in household		
None	2397	27.0 [25.2, 29.0]
1–2 children	5303	59.8 [57.9, 61.7]
3 or more children	1168	13.2 [11.8, 14.7]
Number of household members		
1–3 members	1468	16.6 [15.0, 18.2]
4–6 members	3476	39.2 [37.3, 41.1]
7 or more members	3924	44.2 [42.0, 46.5]
Type of place of residence		
Urban	2364	26.7 [20.6, 33.8]
Rural	5867	66.2 [59.2, 72.5]
Refugee	637	7.2 [5.2, 9.9]
Region		
Central	1804	20.3 [15.9, 25.7]
Eastern	2731	30.8 [26.3, 35.7]
Northern	1419	16.0 [14.1, 18.1]
Western	2914	32.9 [29.4, 36.5]

### 3.2 Distribution of the outcome, exposure, and mediator variables

The results further show that 6143 (69.3%) women slept under an ITN the night before the survey. Approximately 4 in 10 women had exposure to a malaria message, 3334 (37.6%). Approximately 4 in 10 women reported that mosquito bites were the cause of malaria, 3162 (35.7%), while 4031 (45.5%) mentioned mosquitoes as a cause of malaria. Approximately 8 in 10 reported that mosquito bites or mosquitoes were the cause of malaria, 6924 (78.1%). More than 9 in 10 women agreed that malaria can be avoided (8300, 93.6%) and that sleeping under ITN is the best way to prevent malaria (8364, 94.3%). Similarly, more than 9 in 10 women agreed that pregnant women should take antimalarials, 8100 (91.3%); that children should visit the health provider on the same or next day if they developed a fever, 8570 (96.6%); and should receive treatment the same day after developing a fever, 8109 (91.4%). More than 6 in 10 women had comprehensive knowledge of malaria, 5427 (61.2%). Comprehensive knowledge was measured by whether the woman identified the cause of malaria (mosquito bites or mosquitoes as causes of malaria) and answered all remaining items on treatment and prevention correctly. Results are shown in Table 2.

**Table 2:** Distribution of the outcome, exposure, and mediator variables (Weighted sample: *n* = 8868).

Variables	Count	Per cent [95% CI]
Respondent slept under an ITN (Outcome)		
No	2725	30.7 [28.8, 32.8]
Yes	6143	69.3 [67.2, 71.2]
Seen/heard any messages about malaria (Exposure)		
No	5534	62.4 [60.3, 64.5]
Yes	3334	37.6 [35.5, 39.7]
Causes of malaria: Mosquito Bites		
No	5706	64.3 [61.2, 67.4]
Yes	3162	35.7 [32.6, 38.8]
Causes of malaria: Mosquitoes		
No	4837	54.5 [52.2, 56.8]
Yes	4031	45.5 [43.2, 47.8]
Knowledge of Cause (any of the above)		
Don't Know (DK)	1944	21.9 [19.8, 24.2]
Knows Cause	6924	78.1 [75.8, 80.2]
Malaria can be avoided		
No	568	6.4 [5.5, 7.4]
Yes	8300	93.6 [92.6, 94.5]
Sleeping under an ITN is the best way to avoid malaria		
Disagree + DK + uncertain	504	5.7 [4.8, 6.7]
Agree	8364	94.3 [93.4, 95.2]
Pregnant women should take antimalarial medication		
Disagree + DK + uncertain	768	8.7 [7.6, 9.9]
Agree	8100	91.3 [90.1, 92.4]
Children should visit a health provider the same day or the next day after developing a fever		
Disagree + DK + uncertain	298	3.4 [2.8, 4.1]
Agree	8570	96.6 [95.9, 97.2]
How long after developing a fever should a child be taken for treatment?		
Next or other days	759	8.6 [7.6, 9.7]
Same Day	8109	91.4 [90.3, 92.5]
Comprehensive Knowledge of Malaria (Mediator Variable)		
No	3441	38.8 [36.4, 41.3]
Yes	5427	61.2 [58.7, 63.6]

### 3.3 Causal mediation decomposition of malaria prevention message exposure on ITN use

Results in Table 3 indicate that the NIE of exposure to malaria prevention messages mediated through comprehensive malaria knowledge was small and not statistically significant [NIE (average) = 0.001, 95% CI: −0.002, 0.005, *p* = 0.462]. In contrast, the NDE was statistically significant [NDE (average) = 0.064, 95% CI: 0.034, 0.092, *p* < 0.001]. The TE of exposure to malaria prevention messages on ITN use was TE = 0.065, 95% CI: 0.034, 0.094, *p* < 0.001, with only approximately 1.5% [95% CI: −0.033, 0.078, *p* = 0.462] of the effect mediated through comprehensive knowledge of malaria. Sensitivity analysis using E-values showed moderate robustness of the estimated effect to unmeasured confounders.

**Table 3:** Causal mediation decomposition of malaria prevention message exposure on ITN use.

Effect	Estimate	95% CI	*p*-value
Total Effect (TE)	0.065	[0.034, 0.094]	< 0.001
NIE (average)	0.001	[−0.002, 0.005]	0.462
NDE (average)	0.064	[0.034, 0.092]	< 0.001
Proportion Mediated (average)	0.015	[−0.033, 0.078]	0.462

### 3.4 Sensitivity analysis using E-value

Sensitivity analysis using E-values indicated that an unmeasured confounder would need to be associated with both exposure to malaria messages and ITN use by a risk ratio (RR) of at least 1.640 with each association, above and beyond observed covariates, to explain away the observed association fully. To move the CI to include the null (1), a confounder with associations of at least 1.491 for each association would be required. These findings suggest moderate robustness of the estimated effect to unmeasured confounders. Results are presented in Supplementary Table S10.

## 4 Discussion

This study examined whether the effect of exposure to malaria prevention messages on ITN use among women of reproductive age in Uganda was mediated by comprehensive knowledge of malaria. The study found that exposure to malaria prevention messages was associated with a substantial increase in ITN use, with a small proportion of the relationship mediated through comprehensive malaria knowledge. The majority of the effect of malaria prevention messages on ITN use operated through direct pathways independent of comprehensive malaria knowledge. These findings provide important insights into how malaria communication strategies influence preventive behavior, particularly ITN use.

The finding that exposure to malaria prevention messages was associated with increased ITN use is consistent with prior studies that demonstrated that mass media and health promotion communication interventions positively influence malaria prevention behaviors (21, 48). The total effect observed in this study shows that women exposed to malaria prevention messages had a greater likelihood of ITN use than those who were unexposed (49, 50). This evidence suggests that communication campaigns play a key role in malaria-related behavioral interventions across sub-Saharan Africa (51). A key contribution of this study is the decomposition of this total effect into direct and indirect pathways. The indirect effect through comprehensive malaria knowledge was statistically insignificant and small in magnitude, accounting for a limited proportion of the total effect. In contrast, the direct effect of exposure to malaria prevention messages on ITN use remained substantial even after accounting for comprehensive knowledge of malaria. These findings suggest that malaria prevention messages influence ITN use mainly through other mechanisms such as perceived risk, reminders, social norms, motivation, and behavioral cues.

This pattern aligns with behavioral theory, which posits that knowledge alone and information accuracy do not guarantee behavioral change or predict behavior (52–54). Malaria prevention messages may influence behavioral determinants that directly increase ITN use, independent of knowledge gains from exposure. Previous studies reported that exposure to malaria prevention messages can influence ITN use regardless of knowledge levels (21, 28, 48, 55, 56). This may explain why the direct effects of malaria message exposure are substantially higher than those mediated through knowledge of malaria. The small mediated effect through comprehensive malaria knowledge observed in this study may reflect the widespread dissemination of basic malaria knowledge in many settings. In contexts where awareness of malaria transmission and prevention is already high, additional messaging may have limited effect on knowledge but still reinforce behavior. This may explain why the direct behavioral pathway accounted for the majority of the effect observed.

## 5 Study strengths and weaknesses

This study applied a causal mediation framework to decompose the total effect (TE) into natural direct (NDE) and natural indirect (NIE) effects. The consistency of the results across probability and odds ratio scales (Supplementary File S1) suggests a consistent mediation pattern. However, causal interpretation assumes no unmeasured confounding of the exposure–outcome, exposure–mediator, and mediator–outcome relationships. Although we adjusted for several socio-demographic, household, and community factors, residual confounding may remain; for example, ITN access may have preceded exposure to malaria messages and influenced both ITN use and knowledge of malaria. ITN access was not included as a baseline covariate because it may represent a parallel mediating pathway if malaria messages influence ITN acquisition or are delivered alongside net distribution. Therefore, the estimated NDE captures all pathways not operating through knowledge of malaria, potentially including ITN access, and thus should not be interpreted as a purely behavioral effect. The cross-sectional design also limits the ability to establish temporal ordering between exposure, malaria knowledge, and ITN use; longitudinal studies could clarify these relationships. Self-reported exposure to malaria messages and ITN use may also be subject to measurement error and recall bias, though such misclassification is likely non-differential and would bias estimates toward the null, suggesting the observed effects may be conservative.

## 6 Conclusions and recommendations

This study demonstrated that exposure to malaria prevention messages was associated with a higher likelihood of ITN use among women of reproductive age in Uganda. However, only a small proportion of this effect was mediated through comprehensive malaria knowledge, with the majority operating through other pathways, such as behavioral reinforcement and motivational cues, or directly to ITN use. These findings suggest that communication strategies influence preventive behavior beyond knowledge acquisition. Malaria programmes should therefore integrate motivational and behavioral components alongside informational strategies, ensure access to ITNs, and address contextual barriers to consistent use.

## Data Availability

Publicly available datasets were analyzed in this study. This data can be found here: https://dhsprogram.com/data/dataset_admin/index.cfm.
