# Supplementary material for: Does exposure to malaria prevention messages influence insecticide-treated net use through knowledge of malaria? Evidence from a causal mediation analysis among women in Uganda
Source: Front Public Health. 2026 Oct 21;14:1948476. doi: 10.3389/fpubh.2026.1948476 (PMC13731229; doi:10.3389/fpubh.2026.1948476)
Supplement: Supplementary file 1 [file Supplementary_file_1.docx]

**Supplementary File**

**Table 6: Natural Direct, Indirect and Total Effects on the Odds Ratio (OR) Scale**

| **Effect** | **OR** | **95% CI*** |
| --- | --- | --- |
| Natural Direct Effect (NDE) | 1.360 | (1.360, 1.361) |
| Natural Indirect Effect (NIE) | 1.005 | (0.998, 1.013) |
| Total Effect (TE) | 1.368 | (1.358, 1.378) |

*The 95% CIs were based on a 2000 Monte Carlo simulations

**Table 7: Factors associated with comprehensive malaria knowledge**

| **Variable** | **Adjusted OR (95% CI)** | **p-value** |
| --- | --- | --- |
| **Exposure to malaria messages** | | |
| No | 1.00 |  |
| Yes | 1.08 (0.98–1.18) | 0.123 |
| **Age of woman (years)** | | |
| 15–24 | 1.00 |  |
| 25–29 | 1.41 (1.22–1.64) | <0.001 |
| 30 or older | 1.55 (1.32–1.82) | <0.001 |
| **Education level** | | |
| No education | 1.00 |  |
| Primary | 1.31 (1.14–1.50) | <0.001 |
| Secondary or higher | 2.08 (1.76–2.45) | <0.001 |
| **Household wealth quintile** | | |
| Poorest | 1.00 |  |
| Poorer | 1.14 (0.99–1.32) | 0.072 |
| Middle | 1.24 (1.06–1.45) | 0.007 |
| Richer | 1.32 (1.13–1.55) | <0.001 |
| Richest | 1.37 (1.15–1.63) | <0.001 |
| **Number of children under five years** | | |
| None | 1.00 |  |
| 1–2 | 1.23 (1.11–1.37) | <0.001 |
| 3 or more | 1.34 (1.15–1.56) | <0.001 |
| **Sex of household head** | | |
| Male | 1.00 |  |
| Female | 1.11 (1.01–1.23) | 0.034 |
| **Age of household head (years)** | | |
| Younger than 30 | 1.00 |  |
| 30–39 | 0.94 (0.81–1.08) | 0.371 |
| 40–49 | 0.80 (0.69–0.93) | 0.003 |
| 50–59 | 0.78 (0.66–0.91) | 0.002 |
| 60 or older | 0.91 (0.77–1.09) | 0.309 |
| **Parity** | | |
| 0–1 births | 1.00 |  |
| 2–3 births | 1.24 (1.08–1.43) | 0.002 |
| 4 or more births | 1.17 (0.99–1.39) | 0.061 |
| **Region** | | |
| Central | 1.00 |  |
| Eastern | 1.80 (1.57–2.06) | <0.001 |
| Northern | 2.56 (2.15–3.05) | <0.001 |
| Western | 1.76 (1.53–2.03) | <0.001 |

**Note.** OR = odds ratio; CI = confidence interval. Estimates were obtained from the survey-weighted logistic regression mediator model.

**Table 8:** **Factors associated with insecticide-treated net use**

| **Variable** | **Adjusted OR (95% CI)** | **p-value** |
| --- | --- | --- |
| **Exposure to malaria messages** | | |
| No | 1.00 |  |
| Yes | 1.38 (1.25–1.53) | <0.001 |
| **Comprehensive malaria knowledge** | | |
| No | 1.00 |  |
| Yes | 1.43 (1.30–1.57) | <0.001 |
| **Age of woman (years)** | | |
| 15–24 | 1.00 |  |
| 25–29 | 0.96 (0.82–1.13) | 0.635 |
| 30 or older | 1.25 (1.05–1.49) | 0.014 |
| **Education level** | | |
| No education | 1.00 |  |
| Primary | 1.30 (1.12–1.50) | <0.001 |
| Secondary or higher | 1.50 (1.26–1.79) | <0.001 |
| **Parity** | | |
| 0–1 births | 1.00 |  |
| 2–3 births | 1.49 (1.28–1.74) | <0.001 |
| 4 or more births | 1.70 (1.41–2.04) | <0.001 |
| **Household wealth quintile** | | |
| Poorest | 1.00 |  |
| Poorer | 1.17 (1.01–1.37) | 0.040 |
| Middle | 1.20 (1.02–1.42) | 0.031 |
| Richer | 1.38 (1.16–1.64) | <0.001 |
| Richest | 1.57 (1.31–1.89) | <0.001 |
| **Number of children under five years** | | |
| None | 1.00 |  |
| 1–2 | 1.18 (1.05–1.33) | 0.006 |
| 3 or more | 1.13 (0.95–1.35) | 0.176 |
| **Sex of household head** | | |
| Male | 1.00 |  |
| Female | 0.72 (0.65–0.80) | <0.001 |
| **Age of household head (years)** | | |
| Younger than 30 | 1.00 |  |
| 30–39 | 0.99 (0.84–1.17) | 0.945 |
| 40–49 | 0.83 (0.70–0.99) | 0.036 |
| 50–59 | 1.00 (0.82–1.21) | 0.972 |
| 60 or older | 0.64 (0.53–0.78) | <0.001 |
| **Household size** | | |
| 1–3 members | 1.00 |  |
| 4–6 members | 0.76 (0.65–0.89) | <0.001 |
| 7 or more members | 0.59 (0.50–0.70) | <0.001 |
| **Region** | | |
| Central | 1.00 |  |
| Eastern | 0.76 (0.66–0.88) | <0.001 |
| Northern | 1.00 (0.83–1.21) | 0.962 |
| Western | 1.44 (1.23–1.68) | <0.001 |

**Note.** OR = odds ratio; CI = confidence interval; ITN = insecticide-treated net. Estimates were obtained from the survey-weighted logistic regression outcome model.

**Table 9: Potential Outcome Means for Different Mediation Worlds**

| **Quantity** | **Meaning** | **Estimate [95% CI]*** |
| --- | --- | --- |
| μ11 | E[Y(1, M(1))] | 0.734 (0.733, 0.735) |
| μ10 | E[Y(1, M(0))] | 0.733 (0.732, 0.734) |
| μ00 | E[Y(0, M(0))] | 0.668 (0.667, 0.669) |
| μ01 | E[Y(0, M(1))] | 0.670 (0.668, 0.671) |

*The 95% CIs were based on 2000 Monte Carlo simulations

**Table 10: Sensitivity Analysis using E-Value**

| **Quantity** | **Estimate** | **95% CI** |
| --- | --- | --- |
| RR | 1.180 | (1.122, 1.241) |
| E-value | 1.640 | (1.491, NA) |
